# (Bi)sulfite Oxidation by Copper,Zinc-Superoxide Dismutase: Sulfite-Derived, Radical-Initiated Protein Radical Formation

**DOI:** 10.1289/ehp.0901533

**Published:** 2010-03-26

**Authors:** Kalina Ranguelova, Marcelo G. Bonini, Ronald P. Mason

**Affiliations:** 1 Laboratory of Pharmacology, National Institute of Environmental Health Sciences, National Institutes of Health, Department of Health and Human Services, Research Triangle Park, North Carolina, USA; 2 Cardiology, Department of Medicine, University of Illinois at Chicago, Chicago, Illinois, USA

**Keywords:** ESR spin trapping, immuno-spin trapping, sulfite radicals, sulfite toxicity

## Abstract

**Background:**

Sulfur dioxide, formed during the combustion of fossil fuels, is a major air pollutant near large cities. Its two ionized forms in aqueous solution, sulfite and (bi)sulfite, are widely used as preservatives and antioxidants to prevent food and beverage spoilage. (Bi)sulfite can be oxidized by peroxidases to form the very reactive sulfur trioxide anion radical (^•^SO_3_^−^). This free radical further reacts with oxygen to form the peroxymonosulfate anion radical (^−^O_3_SOO^•^) and sulfate anion radical (SO_4_^• −^).

**Objective:**

To explore the critical role of these radical intermediates in further oxidizing biomolecules, we examined the ability of copper,zinc-superoxide dismutase (Cu,Zn-SOD) to initiate this radical chain reaction, using human serum albumin (HSA) as a model target.

**Methods:**

We used electron paramagnetic resonance, optical spectroscopy, oxygen uptake, and immuno-spin trapping to study the protein oxidations driven by sulfite-derived radicals.

**Results:**

We found that when Cu,Zn-SOD reacted with (bi)sulfite, ^•^SO_3_^−^ was produced, with the concomitant reduction of SOD-Cu(II) to SOD-Cu(I). Further, we demonstrated that sulfite oxidation mediated by Cu,Zn-SOD induced the formation of radical-derived 5,5-dimethyl-1-pyrroline *N*-oxide (DMPO) spin-trapped HSA radicals.

**Conclusions:**

The present study suggests that protein oxidative damage resulting from (bi)sulfite oxidation promoted by Cu,Zn-SOD could be involved in oxidative damage and tissue injury in (bi)sulfite-exacerbated allergic reactions.

Sulfur dioxide is one of the major atmospheric pollutants, but its two ionized forms in aqueous solution at neutral pH, sulfite (SO_3_^2−^) and (bi)sulfite (HSO_3_^−^), are widely used as antioxidants and preservatives in beverages and foods (Danilewicz 2003). However, the prevalence of sulfite toxicity is relatively high, and it has been associated with allergic reactions characterized by sulfite sensitivity, asthma, and anaphylactic shock (Komarnisky et al. 2003). Sensitive individuals can experience such adverse reactions when they consume sulfites, with asthmatics being particularly vulnerable to such toxicity.

Sulfite is detoxified in the liver and lung to sulfate by sulfite oxidase, a molybdenum-dependent mitochondrial enzyme (Cohen and Fridovich 1971); sulfite oxidase deficiency is one of the most accepted causes of sulfite hypersensitivity and toxicity. This enzymatically catalyzed oxidation has been shown to proceed via a two-electron oxidation without the formation of any detectable radical intermediates. In contrast, recent studies suggest that the cytotoxicity of (bi)sulfite is mediated by free radicals, because (bi)sulfite increases reactive oxygen species formation, and antioxidants and free radical scavengers prevent its toxicity (Niknahad and O’Brien 2008). In addition, transition metals catalyze the autoxidation of (bi)sulfite via sulfur trioxide anion radical (^•^SO_3_^−^) formation:


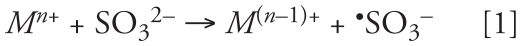


where *M* may be copper (Cu^2+^), iron (Fe^3+^), oxivanadium anion (VO^2+^), manganese (Mn^2+^), nickel (Ni^2+^), or chromate anion (CrO_4_^2 −^) (Alipazaga et al. 2004; Berglund et al. 1993; Brandt and Elding 1998; Lima et al. 2002; Shi 1994), but this reaction requires higher concentrations of (bi)sulfite to permit effective propagation of the chain reaction. In a recent study, Alipazaga et al. ( 2009) reported oxidative DNA damage induced by (bi)sulfite solutions in the presence of Cu(II) peptide complexes. It has also been shown that free radicals have been produced by enzymatic initiation of the oxidation of (bi)sulfite by prostaglandin H synthase (Mottley et al. 1982a) and horseradish peroxidase (HRP) (Araiso et al. 1976; Mottley et al. 1982b), with formation of ^•^SO_3_^−^. This predominantly sulfur-centered radical (Chantry et al. 1962) reacts with molecular oxygen by forming the peroxymonosulfate anion radical (^−^O_3_SOO^•^), which is a precursor of the sulfate anion radical (SO_4_^• −^) (Neta et al. 1988):


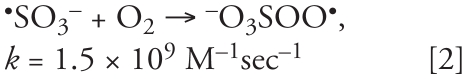



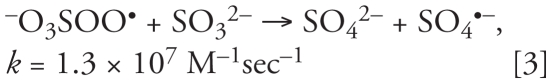



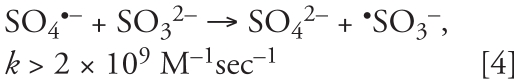


SO_4_^• −^ is a very strong oxidant, nearly as strong as the hydroxyl radical, and it is very likely to oxidize other biomolecules by one-electron oxidation.

It is possible that bisulfite may also lead to further reactive sulfur species via the peroxidase activity of enzymes such as copper,zinc-superoxide dismutase (Cu,Zn-SOD), a metalloenzyme that catalyzes the dismutation of the superoxide anion into O_2_ and hydrogen peroxide (H_2_O_2_). At high pH, Cu,Zn-SOD exhibits peroxidase activity, with the initial step of the peroxidase cycle being a reduction of SOD-Cu(II) by H_2_O_2_ or its deprotonated form, HO_2_^−^, to SOD-Cu(I) (Bonini et al. 2009; Fuchs and Borders 1983; Hodgson and Fridovich 1973). At neutral pH, the peroxidase activity of Cu,Zn-SOD is stimulated in the presence of bicarbonate (HCO_3_^−^) buffer (Bonini et al. 2004; Liochev and Fridovich 2004; Zhang et al. 2000). It has been proposed that at pH 7.4, anions structurally similar to HCO_3_^−^, such as (bi)sulfite (HSO_3_^−^) and (bi)selenite (HSeO_3_^−^), may also stimulate the peroxidase activity of Cu,Zn-SOD in the presence of millimolar H_2_O_2_ (Sankarapandi and Zweier 1999).

In the present study, we evaluated the role of Cu,Zn-SOD in (bi)sulfite oxidation and found that, under our experimental conditions, SOD1-Cu(II) is slowly reduced to SOD1-Cu(I) by (bi)sulfite. We used optical spectroscopy, electron spin resonance (ESR), and oxygen uptake experiments to demonstrate that (bi)sulfite (as Na_2_SO_3_) was a one-electron donor substrate for Cu,Zn-SOD, leading to the generation of reactive sulfur radicals via Equations 2–4. We also applied immuno-spin trapping with 5,5-dimethyl-1-pyrroline *N*-oxide (DMPO) to investigate oxidation of target proteins [e.g., human serum albumin (HSA) at plasma levels] to protein radicals (Figure 1). We found that (bi)sulfite oxidation mediated by Cu,Zn-SOD generated the formation of HSA radicals, which might be responsible for the tissue injury in allergic reactions to (bi)sulfite.

## Materials and Methods

### Chemicals

We purchased bovine kidney superoxide dismutase (SOD) from Calzyme Laboratories Inc. (San Luis Obispo, CA). HSA (99.99% purity), diethylenetriaminepentaacetic acid (DTPA), sodium sulfite, thiocyanate, azide, cyanide, and H_2_O_2_ (obtained as a 30% solution) were from Sigma Chemical Co. (St. Louis, MO). We determined the H_2_O_2_ concentration from its absorbance at 240 nm (ɛ = 39.4 M ^−1^cm ^−1^). DMPO (high purity, ≥ 99%) from Alexis Biochemicals (San Diego, CA) was sublimed twice under vacuum at room temperature and stored under an argon atmosphere at −80°C before use. Chelex-100 resin was from Bio-Rad Laboratories (Hercules, CA).

### ESR spectroscopy

We obtained ESR spin-trapping data at room temperature using a Bruker EMX spectrometer with 100 kHz modulation frequency and equipped with an ER 4122 SHQ cavity (Bruker BioSpin Corp., Billerica, MA). We placed samples in a 10-mm flat cell (200 μL final volume) and initiated recording of the spectra within 1 min of the start of the reaction.

We recorded low-temperature ESR data at 77 K after the indicated incubation times. Initially, we mixed SOD with (bi)sulfite at room temperature; after incubation, we transferred the reaction mixtures into 1-mL polyethylene syringes and froze them in liquid nitrogen. We added glycerol (10%) to the samples before freezing to prevent cracking of the frozen texture.

### Oxygen uptake

For oxygen uptake measurements, we added 500 μL sodium sulfite to a chamber equipped with a Clark electrode and a stirrer. We initiated the reaction (1.8 mL) by SOD, and the oxygen uptake curves were obtained at room temperature with an oxygen monitor (model 53; Yellow Springs Instrument Co., Yellow Springs, OH).

### Chemical reactions

Typically, we carried out reactions of 600 μM HSA, 500 μM Na_2_SO_3_, and 50 μM Cu,Zn-SOD in the presence or absence of 5 mM DMPO in 100 mM phosphate buffer (Chelex-treated with 25 μM DTPA) at pH 7.4 in a total volume of 30 μL. After 1 hr of incubation at 37°C, we stopped reactions with 5 mM reduced glutathione and then diluted the samples with deionized H_2_O for electrophoresis and immuno-spin trapping analyses.

### Coomassie blue stain, Western blot, and ELISA (enzyme-linked immunosorbent assay)

We electrophoresed the reaction mixtures under reducing conditions through duplicate 4–12% BisTris NuPage acylamide gels (Invitrogen, Carlsbad, CA). We performed Western blotting and ELISA analysis as previously described (Detweiler et al. 2002) with minor changes (we used fish gelatin instead of casein to prevent the nonspecific binding sites).

### Optical spectroscopy

We recorded optical data on a Cary 100 spectrophotometer (Varian Inc., Palo Alto, CA) using a 500 μM quartz cuvette. We determined Cu,Zn-SOD concentration from the broad band at 680 nm (ɛ = 300 M^−1^cm^−1^ in the bovine enzyme), which results from the d-d transitions of the Cu atom (Foti et al. 1997). We carried out reactions in 100 mM phosphate buffer at pH 7.4. Cu,Zn-SOD (1 mM) was added first, followed by 20 mM (bi)sulfite, and each scan was recorded every 3 min for 30 min.

## Results

### (Bi)sulfite oxidation by Cu,Zn-SOD detected by optical spectroscopy, ESR, and oxygen uptake

When we added a 20-fold excess of (bi)sulfite to 1 mM Cu,Zn-SOD, the absorption band at 680 nm characteristic of the active site of SOD1-Cu(II) decreased slowly, then completely disappeared as the wild-type protein was reduced to Cu(I) (Figure 2A). We recorded the optical spectra every 3 min, and within < 30 min we observed a full reduction of Cu(II) to Cu(I) by (bi)sulfite. However, lower concentrations of protein (50 μM) and (bi)sulfite (500 μM) were sufficient for low-temperature ESR spectra to detect the reduction of Cu(II) in Cu,Zn-SOD (Figure 2B). ESR data showed that the addition of a 10-fold excess of (bi)sulfite to Cu,Zn-SOD followed by a 1-hr incubation resulted in an approximately 40% decrease in ESR intensity compared with the untreated protein (Figure 2B). The anisotropic hyperfine coupling constant (A_||_ = 135 G) remained unchanged during the incubation time, indicating that (bi)sulfite does not bind directly to the active site Cu(II) (Strothkamp and Lippard 1981).

To determine whether (bi)sulfite is oxidized by Cu(II) in Cu,Zn-SOD, we also performed room-temperature ESR spin-trapping experiments. When we mixed Cu,Zn-SOD (50 μM) with (bi)sulfite (500 μM) in the presence of the spin trap DMPO (100 mM), it generated an intense ESR signal (Figure 3A, spectrum a) corresponding to the assigned ^•^SO_3_^−^ adduct of DMPO, DMPO/^•^SO_3_^−^ (*a*^H^_β_= 16.0 G and *a*^N^ = 14.7 G) (Mottley and Mason 1988; Mottley et al. 1982a, 1982b). Previous studies have shown that (bi)sulfite stimulates the peroxidase function of Cu,Zn-SOD and that ^•^SO_3_^−^ is formed when the protein is treated with 1 mM H_2_O_2_ in the presence of 20 mM (bi)sulfite (Sankarapandi and Zweier 1999). According to the authors, control experiments in the absence of Cu,Zn-SOD confirmed that the ^•^SO_3_^−^ signal was not due to direct oxidation of (bi)sulfite by H_2_O_2_, which is known from the literature (Flockhart et al. 1971; Mottley et al. 1982a) to proceed nonenzymatically at high concentrations of H_2_O_2_ via the following reaction:


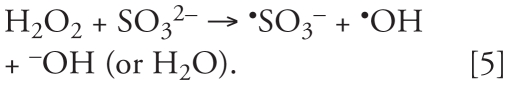


To determine the effect of low and nontoxic concentration of H_2_O_2_ (100 μM) and to confirm that ^•^SO_3_^−^ is generated because of the enzymatic oxidation of (bi)sulfite, we performed control experiments in the presence and absence of H_2_O_2_. Contrary to expectation (Sankarapandi and Zweier 1999), addition of 100 μM H_2_O_2_ had almost no effect on the ESR intensity of DMPO/^•^SO_3_^−^ (Figure 3A, spectra a and b), and omission of (bi)sulfite (Na_2_SO_3_) or Cu,Zn-SOD resulted in no radical adduct formation (Figure 3A, spectra c and d, respectively). Control experiments confirmed that the reaction is insensitive to catalase, implying that H_2_O_2_ is not involved (data not shown).

The proposed mechanism of enzymatic oxidation of (bi)sulfite to ^•^SO_3_^−^ by the active Cu(II) site of Cu,Zn-SOD proceeds in a one-electron reduction reaction of Cu(II) by (bi)sulfite, similar to the oxidation of (bi)sulfite by HRP and prostaglandin H synthase (Mottley and Mason 1988; Mottley 1982a, 1982b; Roman and Dunford 1973). The resulting ^•^SO_3_^−^ is known to react further with molecular oxygen to form ^−^O_3_SOO^•^ and SO_4_^• −^ in the free radical chain mechanism previously reported (Hayon et al. 1972; Mottley and Mason 1988; Reed et al. 1986). To confirm our hypothesis, we next investigated the consumption of oxygen by 500 μM (bi)sulfite, with the reaction initiated by 0–500 μM Cu,Zn-SOD. When ^•^SO_3_^−^ reacted with oxygen in the absence of spin trap, we observed oxygen consumption strongly dependent on the Cu,Zn-SOD concentration (Figure 3B). Addition of 500 μM Cu,Zn-SOD resulted in approximately 30% oxygen consumption after 15 min. When we examined the effect of the spin trap DMPO using 500 μM Na_2_SO_3_ and 500 μM Cu,Zn-SOD as the initiator, prior or later additions of 100 mM DMPO (the same amount used for the spin-trapping ESR data) almost completely prevented oxygen uptake (Figure 3B), that is, no radical chain reactions ended in the formation of ^−^O_3_SOO^•^ and SO_4_^• −^.

To characterize the importance of Cu redox cycling at the enzyme-active site upon the generation of ^•^SO_3_^−^, we mixed 500 μM Na_2_SO_3_ with selected inhibitors and initiated the reactions by 50 μM Cu,Zn-SOD in the presence of 100 mM DMPO (Figure 4). The ESR intensity of the spectra showed that addition of 500 μM thiocyanate, azide, or cyanide in the presence of an equimolar amount of (bi)sulfite significantly inhibited ^•^SO_3_^−^ production. These results strongly suggest that because these anions bind directly to the Cu with high affinity, the enzymatic activity of Cu,Zn-SOD is inhibited, and no further oxidation of (bi)sulfite to sulfite-derived radicals is possible.

### Formation of HSA-DMPO nitrone adducts induced by the Cu,Zn-SOD-(bi)sulfite system as determined by immuno-spin trapping

The optical and ESR data showed that (bi)sulfite is oxidized by Cu,Zn-SOD to ^•^SO_3_^−^, which will initiate the radical chain reaction with formation of ^−^O_3_SOO^•^ and SO_4_^• −^ via Equations 2–4. To characterize the ability of these radicals to oxidize amino acid(s) in target proteins, we incubated HSA with the enzyme and (bi)sulfite in the presence of DMPO and analyzed the reaction products by Western blotting using an anti-DMPO polyclonal antibody (Detweiler et al. 2002). We chose a concentration of DMPO that was much less than the 100 mM used for the ESR and oxygen uptake, so as to not inhibit the chain reaction yet be sufficient for the protein radicals to react with DMPO for detection by anti-DMPO antibody. We achieved the overall high yield of protein DMPO nitrone adducts by decreasing the DMPO concentration to 5 mM in the presence of the plasma concentration of HSA (600 μM). We mixed samples containing 600 μM HSA with 500 μM Na_2_SO_3_ in the presence of 5 mM DMPO and initiated the reactions with 10, 20, 30, 40, and 50 μM Cu,Zn-SOD. Coomassie blue staining of the gel verified the amount of HSA present in all treatments and showed the presence of a single band at 60 kDa, which corresponds to the size of albumin, together with a small amount of HSA dimer at approximately 120 kDa (Figure 5A). We also detected a very weak band at approximately 15 kDa at a Cu,Zn-SOD concentration of 50 μM, corresponding to its monomer. We performed immunochemical detection of HSA–DMPO nitrone adducts using Western blotting and ELISA in parallel with SDS-PAGE. As shown in Figure 5B, samples lacking Cu,Zn-SOD, DMPO, or Na_2_SO_3_ contained negligible anti-DMPO cross- reacting material. Incubation of HSA with > 10 μM Cu,Zn-SOD resulted in a significant increase in HSA-DMPO–derived nitrone adducts as assessed by ELISA (Figure 5C). This result, together with the oxygen uptake experiments, demonstrated that 5 mM DMPO, because it did not trap the entire primary ^•^SO_3_^−^, allowed the radical chemistry in Equations 2–4 to proceed with the formation of the damaging radical intermediates.

HSA-derived nitrone adducts also depended on the (bi)sulfite concentration (Figure 6A). Omission of HSA, DMPO, (bi)sulfite, or Cu,Zn-SOD resulted in no immunostaining above the background level. When 0.1 mM (bi)sulfite and 600 μM albumin were oxidized in the presence of 5 mM DMPO and 50 μM Cu,Zn-SOD, we detected a faint band of DMPO–nitrone adducts. Western blotting performed on reactions containing 0.25–3 mM (bi)sulfite showed increased production of DMPO-HSA radical-derived nitrone adducts and very weak bands of DMPO-HSA dimer at the higher (bi)sulfite concentrations.

We also determined the effect of time on the formation of HSA radical-derived nitrone adducts (Figure 6B,C). In the presence of 5 mM DMPO, 500 μM Na_2_SO_3_, and 50 μM Cu,Zn-SOD, Western blotting showed that DMPO-HSA radical-derived nitrone adduct production increased with reaction time, reaching saturation at about 1 hr. ELISA data paralleled those from Western blotting (Figure 6C).

## Discussion

**Figure 1:**
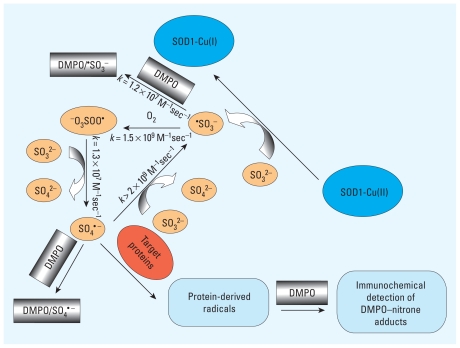
Proposed mechanism of protein oxidative damage induced by the Cu,Zn-SOD–(bi)sulfite system.

**Figure 2:**
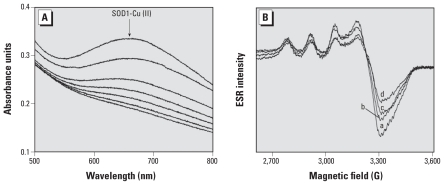
(*A*) Reduction of Cu,Zn-SOD by (bi)sulfite; optical spectra were observed from 1 mM Cu,Zn-SOD in phosphate buffer (100 mM, pH 7.4) in the presence of 20 mM Na_2_SO_3_ and were recorded every 3 min. (*B*) Effect of (bi)sulfite on the ESR spectra of the active-site Cu^2+^ of SOD. Spectra were observed from 50 μM Cu,Zn-SOD in 100 mM phosphate buffer, pH 7.4, at 77 K; incubations were performed at 37°C, and the samples were frozen in liquid nitrogen. Spectra were recorded with 0.5 mM (bi)sulfite at different time intervals: spectrum a, 1 min; spectrum b, 15 min; spectrum c, 30 min; spectrum d, 60 min. Instrumental parameters were as follows: microwave frequency, 9.50 GHz; microwave power, 2 mW; modulation amplitude, 4 G; receiver gain, 5 × 10^4^; and scan rate, 9 G/sec. Each spectrum is a single scan.

**Figure 3:**
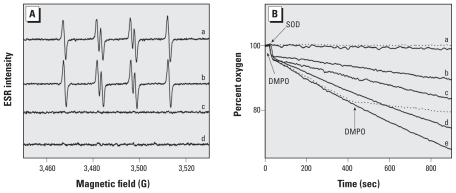
(*A*) ESR spectra of DMPO/^•^SO_3_^−^ generated by Cu,Zn-SOD and (bi)sulfite. Spectrum a was detected by mixing 50 μM Cu,Zn-SOD, 100 mM DMPO, 500 μM Na_2_SO_3_, and 100 μM H_2_O_2_ in 100 mM phosphate buffer, pH 7.4, and then recorded immediately at room temperature. Spectra b–d are the same as spectrum a but without H_2_O_2_ (spectrum b), without Na_2_SO_3_ (spectrum c), or without SOD (spectrum d). Instrumental parameters were as follows: microwave frequency, 9.81 GHz; microwave power, 20 mW; modulation amplitude, 0.5 G; receiver gain, 5 × 10^4^; scan rate, 0.5 G/sec. Each spectrum is a single scan. (*B*) Oxygen uptake curves as a function of Cu,Zn-SOD concentration. Sodium (bi)sulfite (Na_2_SO_3_, 500 μM) was placed in a chamber in 100 mM phosphate buffer, pH 7.4, and the reaction was initiated with different concentrations of Cu,Zn-SOD: spectrum a, 0 μM; spectrum b, 50 μM; spectrum c, 100 μM; spectrum d, 300 μM; spectrum e, 500 μM. The uptake curves were the same as spectrum e but with 100 mM DMPO added before (upper dotted line) or 400 sec after (lower dotted line) the addition of Cu,Zn-SOD.

**Figure 4:**
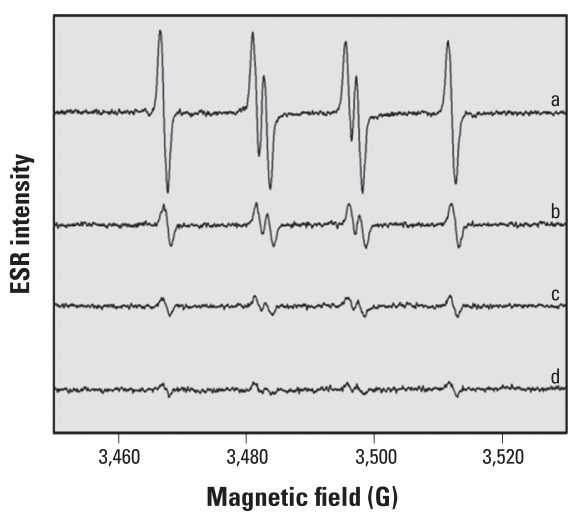
Effect of Cu,Zn-SOD inhibitors on the ESR intensity of DMPO/^•^SO_3_^−^ adducts. Spectrum a was observed from Cu,Zn-SOD (50 μM), Na_2_SO_3_ (500 μM), and DMPO (100 mM) in phosphate buffer (100 mM, pH 7.4) and recorded immediately at room temperature. Spectra b–d are the same as spectrum a except with 500 μM of sodium thiocyanate (spectrum b), sodium azide (spectrum c), or sodium cyanide (spectrum d) added to the phosphate buffer. Instrumental parameters were as follows: microwave frequency, 9.81 GHz; microwave power, 20 mW; modulation amplitude, 0.5 G; receiver gain, 5 × 10^4^; scan rate, 0.5 G/sec.

**Figure 5:**
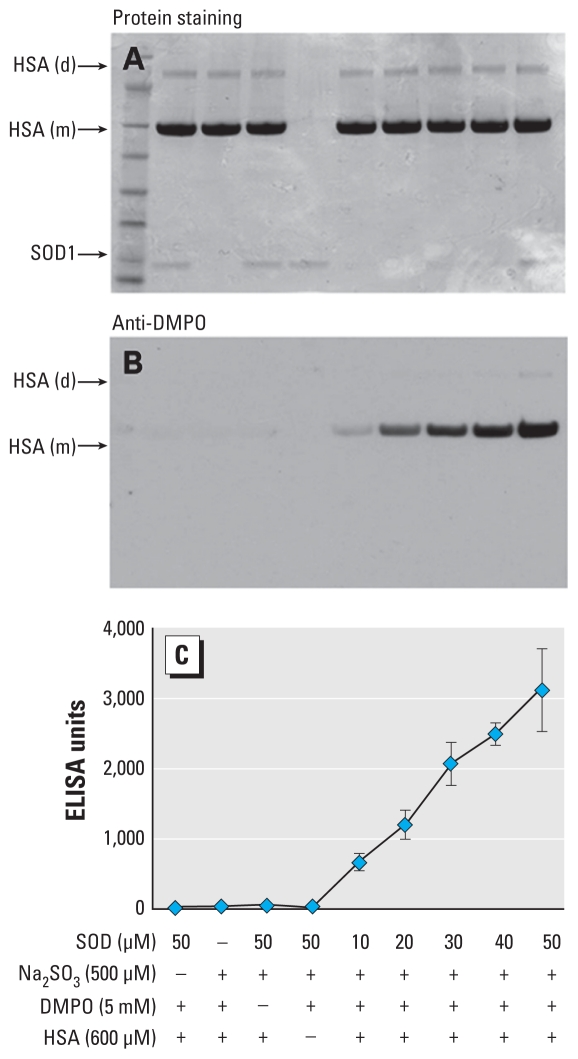
Concentration-dependent effects of Cu,Zn-SOD on the formation of HSA radical-derived nitrone adducts shown by (*A*) Coomassie blue staining, (*B*) anti-DMPO Western blotting, or (*C*) ELISA analysis. Abbreviations: d, dimer; m, monomer. Reactions including HSA (600 μM), Na_2_SO_3_ (500 μM), and DMPO (5 mM) were initiated with Cu,Zn-SOD as indicated, and the mixtures were incubated for 1 hr at 37°C in 100 mM phosphate buffer (pH 7.4). Each lane contained 3.8 μg HSA.

**Figure 6:**
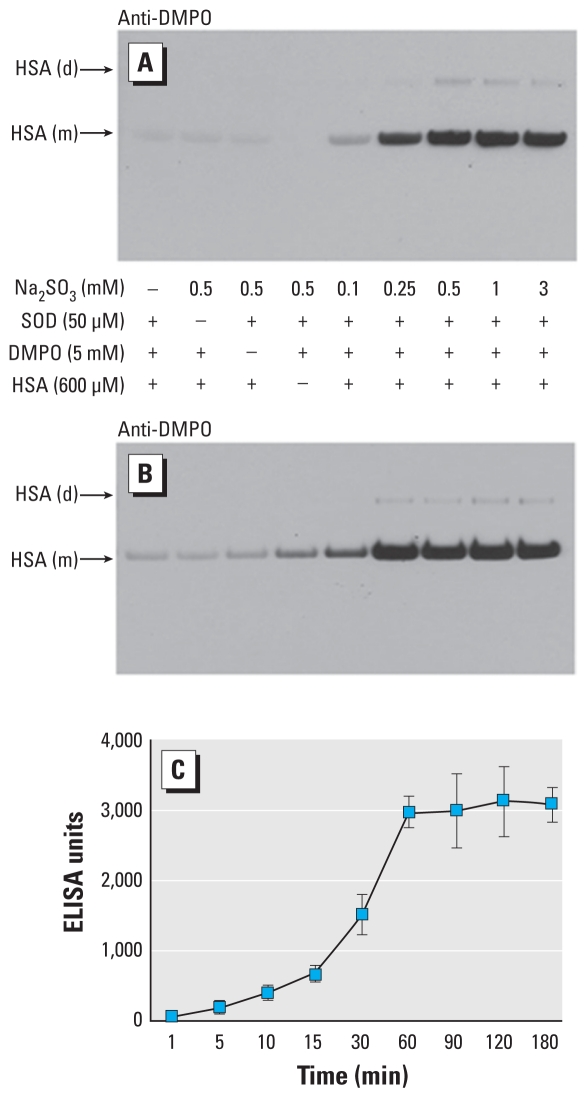
(*A*) Concentration-dependent effects of Na_2_SO_3_ on the formation of HSA radical-derived nitrone adducts. Reactions—including HSA (600 μM), DMPO (5 mM), and (bi)sulfite, as indicated—were initiated with 50 μM SOD, and the mixtures were incubated for 1 hr at 37°C in 100 mM phosphate buffer (pH 7.4). Abbreviations: d, dimer; m, monomer. (*B*) Effect of time on the formation of HSA radical-derived nitrone adducts by anti-DMPO immunostain. (*C*) ELISA analysis. Reactions containing HSA (600 μM), DMPO (5 mM), and Na_2_SO_3_ (500 μM) were initiated with 50 μM SOD, the mixtures were incubated at 37°C in 100 mM phosphate buffer (pH 7.4), and reactions were stopped with 5 mM reduced glutathione at the time points indicated. Each lane contained 3.8 μg HSA.

The present data confirm that the enzymatic oxidation of (bi)sulfite by Cu,Zn-SOD proceeds via a radical mechanism as demonstrated using optical spectroscopy, oxygen uptake, and ESR experiments. Similar results have been reported for some peroxidases (e.g., HRP, prostaglandin H synthase) (Araiso et al. 1976; Mottley et al. 1982a, 1982b). Once the (bi)sulfite is oxidized by Cu(II) in Cu,Zn-SOD and ^•^SO_3_^−^ is formed, it reacts very rapidly with oxygen and generates ^−^O_3_SOO^•^ and SO_4_^• −^ (Hayon et al. 1972), which—as very powerful oxidants (*E*^−^_O^3^SOO_^•^_/_^−^_O^3^SOOH_ = 1.1 V, *E*_SO^4^_^• −^_/SO^4^_^2−^ = 2.43 V)—can attack target proteins (e.g., HSA in plasma) (Neta et al. 1988; Steele and Appelman 1982) (Figure 1). Previous work on the oxidation of (bi)sulfite by the HRP–H_2_O_2_ system and ESR spin-trapping experiments showed that there is a strong competition between the spin trap DMPO and oxygen for ^•^SO_3_^−^ (Ranguelova and Mason 2009). In fact, in the latter system, the formation of the oxygen-derived radicals ^−^O_3_SOO^•^ and SO_4_^• −^ was almost prevented by high DMPO concentrations (100 mM) (Figure 3B), and a decrease of the spin-trap concentration to ≤ 3 mM was required to trap protein radicals formed by ^−^O_3_SOO^•^ and SO_4_^• −^ (Mottley and Mason 1988). The very slow consumption of oxygen observed even in the presence of 100 mM DMPO is likely due to the rapid reaction of ^•^SO_3_^−^ with oxygen at a diffusion-controlled rate to form ^−^O_3_SOO^•^, which then reacts with SO_3_^2−^ to produce SO_4_^• −^ (Figure 1).

(Bi)sulfite is one of the few sulfating agents approved by the Food and Drug Administration as a food preservative and antioxidant to prevent or reduce spoilage (Gunnison 1981). However, sulfites have been associated with adverse allergic and asthmatic reactions experienced by sulfite-hypersensitive individuals. The most frequent sulfite-reaction symptoms are difficulty in breathing, food intolerance symptoms, asthma, and occasionally anaphylactic shock. There is no specific treatment for sulfite toxicity, and in general, to our knowledge, the mechanisms of the potentially toxic reactions of (bi)sulfite are poorly understood.

One reason for the toxic potential of (bi)sulfite is a deficiency of sulfite oxidase, the molybdenum-containing enzyme that oxidizes sulfite to sulfate (SO_4_^2−^), and it is noteworthy that in cases of sulfite oxidase deficiency, the concentration of sulfite in plasma is abnormal (> 1 mM) (Acosta et al. 1989; Johnson et al. 1980). The capacity of sulfite oxidase for sulfite oxidation is extremely high, with the reaction proceeding via a one-step, two-electron oxidation to sulfate with no free radical intermediates (Cohen and Fridovich 1971). However, Yokoyama et al. (1971) showed that inhaled sulfur dioxide does reach the blood plasma, where the dissolved SO_2_ [(bi)sulfite] forms oxidation products other than sulfate, such as S-sulfonates (Bechtold et al. 1993); this indicates the presence of another mechanism of (bi)sulfite oxidation besides the well-known sulfite oxidase route. Another radical mechanism of xanthine-dependent aerobic oxidation of (bi)sulfite in the presence of xanthine oxidase has been proposed by McCord and Fridovich (1968). The authors concluded that xanthine oxidase, when catalyzing the aerobic oxidation of xanthine, generated a superoxide anion, which then served to initiate the (bi)sulfite chain reaction. A previous report from our laboratory (Mottley et al. 1982b) demonstrated that incubation of (bi)sulfite with HRP and H_2_O_2_ is not sensitive to the presence of SOD, confirming that the peroxidase-catalyzed pathway does not involve a superoxide chain reaction.

In the present study we used Cu,Zn-SOD^−^ (bi)sulfite as a source for generation of oxidants (^−^O_3_SOO^•^ and SO_4_^• −^) that are diffusible and radicals themselves to show their capability to oxidize the most abundant plasma protein (albumin) to protein radicals (Figure 1). Our Western blot experiments showed that in the presence of DMPO, the Cu,Zn-SOD–(bi)sulfite system produced sulfite-derived radicals that oxidized albumin to produce protein-centered radicals trapped by the nitrone spin-trap DMPO and detected as DMPO-HSA nitrone adducts. When DMPO or any of the other system components were eliminated, no immunostaining appeared above the background signal levels, confirming that all of the reactants are needed for detection of radicals. The extent of immuno-spin trapping increased with spin-trap concentrations up to 10 mM and then decreased (data not shown). These results are consistent with the oxygen uptake experiments discussed above and with the ESR data for SO_4_^• −^ (Mottley and Mason 1988), showing that lower concentrations of the spin trap must be used so that all the primary radicals are not trapped but have a chance for further reaction. Moreover, recent studies have confirmed the ability of DMPO to trap different protein radicals from the same system by varying its concentration (Bhattacharjee et al. 2007). Production of HSA nitrone adducts was also dependent on (bi)sulfite and Cu,Zn-SOD concentrations; only 500 μM (bi)sulfite was sufficient to detect positive results on the anti-DMPO Western blots, whereas (bi)sulfite concentration in wines, where it is used as a preservative, is 6 mM (Gunnison 1981).

In summary, our study showed that Cu,Zn-SOD^−^ (bi)sulfite provides an enzymatic pathway to generate the reactive intermediates ^−^O_3_SOO^•^ and SO_4_^• −^, which oxidize HSA residues to protein radicals. We also propose that Cu,Zn-SOD may contribute to oxidative damage and tissue injury in (bi)sulfite (sulfur dioxide)–exacerbated allergic reactions. Our results suggest that SOD-dependent, sulfite-mediated oxidation of albumin residues is likely to occur *in vivo*, particularly at sites where Cu,Zn-SOD concentration is higher. Further studies are necessary to clarify whether alterations in Cu,Zn-SOD activity affect (bi)sulfite toxicity.
